# Nucleotide Variant in the *SLC26A9* Gene in Two Siblings with Cystic Fibrosis

**DOI:** 10.3390/jcm15114067

**Published:** 2026-05-25

**Authors:** Adam Krusiński, Anna Grenda, Adrian Obara, Irena Węgrzyn-Szkutnik, Wojciech Zygmunt, Hanna Winiarska, Barbara Kuźnar-Kamińska, Łukasz Gajek, Jan Siwiec, Paweł Krawczyk, Janusz Milanowski

**Affiliations:** 1 Department of Pneumonology, Oncology and Allergology, Medical University of Lublin, Jaczewskiego 8, 20-950 Lublin, Poland; irena.szkutnik@gmail.com (I.W.-S.);; 2 Laboratory of Immunology and Genetics, Medical University of Lublin, Jaczewskiego 8, 20-950 Lublin, Poland; 3 Institute of Genetics and Immunology Genim LCC, Filaretów 27/2, 20-609 Lublin, Poland; obara12lb@interia.pl (A.O.);; 4 Department of Pulmonology, Allergology and Respiratory Oncology, Poznan University of Medical Sciences, 61-701 Poznań, Poland

**Keywords:** cystic fibrosis, *CFTR* gene, *SLC26A9* gene

## Abstract

**Background:** Currently, increasing attention is being paid to the role of genes other than *CFTR* and their variants as factors modifying the course of cystic fibrosis (CF). One such gene is *SLC26A9*, which encodes a protein involved in chloride and bicarbonate transport across the epithelial cell membrane. Variants of *SLC26A9*, such as c.229G>A (p.Gly77Ser) and c.1885C>T (p.Pro629Ser), have been described in patients with severe and rapidly progressive CF. The aim of this study was to identify *SLC26A9* variants in a group of 20 patients with CF. **Methods:** DNA was isolated from blood samples and collected from all patients. Fragments of exons 3 and 17 of the *SLC26A9* gene were amplified by PCR and sequenced using the Sanger method. **Results:** An SLC26A9 variant was identified in two siblings. These patients were diagnosed with CF in adulthood and presented with moderate pulmonary symptoms without exocrine pancreatic insufficiency. In both siblings carrying the CFTR variants p.Phe508del and c.3140-26A>G, the *SLC26A9* variant c.1847C>T (p.Pro616Leu) was detected. This variant has not been widely described in the literature and has not previously been associated with CF. **Conclusions:** The c.1847C>T (p.Pro616Leu) variant is located near a domain that may affect the transport function of the SLC26A9 protein. However, patients in whom the variant was identified did not present a severe disease phenotype. Further studies on larger patient cohorts are required, and at present this variant should be considered of uncertain significance in CF.

## 1. Introduction

Cystic fibrosis (CF) is an autosomal recessive disorder caused by pathogenic variants in the *CFTR* (CF transmembrane conductance regulator) gene, most frequently the c.1521_1523del (p.Phe508del) variant, present in either homozygous or heterozygous form. CFTR protein is a membrane chloride and bicarbonate channel in epithelial cells. Pathogenic variants result in the absence of the protein or in altered structure or function. Impaired ion transport leads to abnormal physicochemical properties of exocrine gland secretions. The primary manifestation of CF is pulmonary disease characterized by impaired mucociliary clearance, increased inflammation, and chronic lower respiratory tract infections caused by bacteria such as *Pseudomonas aeruginosa* or *Staphylococcus aureus*. These complications eventually lead to respiratory failure and premature death [1,2].

The CFTR protein is not the only ion channel present in the epithelial cell membrane. Its dysfunction affects the activity of other transport proteins, particularly those involved in sodium and potassium ion transport. Of particular interest are the chloride and bicarbonate channels encoded by *SLC26A9* (solute carrier family 26 member 9) channels, which are expressed, among others, in the epithelium of the respiratory and gastrointestinal tracts [3,4,5]. It is hypothesized that the normal protein may partly compensate for CFTR dysfunction in patients with cystic fibrosis. Moreover, variants in the *SLC26A9* gene have been associated with clinical symptoms similar to those observed in CF, including bronchiectasis, diabetes, and even meconium ileus. Nucleotide changes in *SLC26A9* may contribute to a more severe disease course, and both the gene and its protein product may represent potential targets for therapeutic intervention.

In 2024, Shiyu Luo and colleagues identified two variants in the *SLC26A9* gene in three of the six patients with severe and rapidly progressive CF, i.e., c.229G>A (p.Gly77Ser, exon 3) and c.1885C>T (p.Pro629Ser, exon 17). In genetic databases, these are classified as variants of uncertain significance (VUS). The authors concluded that impairment of SLC26A9 protein function exacerbated the clinical course of CF by further disrupting the ion transport mediated by both CFTR and SLC26A9 proteins [6,7]. Our study, combining a case-based observational approach with molecular analysis, aimed to analyze the specific SLC26A9 gene regions identified in the aforementioned publication. The study included patients with CF treated at the Department of Pneumonology, Oncology and Allergology of the University Clinical Hospital No. 4 in Lublin (Poland).

## 2. Methodology

### 2.1. Patients

Twenty adult patients with CF treated at the University Clinical Hospital No. 4 in Lublin, Poland, were enrolled in the study. During the diagnostic workup, pathogenic variants were identified on both alleles of the *CFTR* gene in all patients. Twelve study subjects were homozygous for the p.Phe508del variant, while the remaining individuals were compound heterozygotes, carrying the p.Phe508del variant in combination (in trans) with other *CFTR* variants. Moreover, patients heterozygous for the p.Phe508del and p.Ser670_Leu671insTer variants were also carriers of the IVS-8-polyT polymorphism. All identified variants are summarized in Table 1.

All patients suffered from a severe form of CF with bronchopulmonary disease, which, in most cases, was also comorbid with exocrine pancreatic insufficiency.

The study was approved by the Bioethics Committee at the Medical University of Lublin (Approval No. KB-0024/166/12/2024). Informed consent was obtained from all participants. Experiments were performed in accordance with relevant guidelines, including the Declaration of Helsinki.

### 2.2. DNA Isolation

Peripheral blood was collected in EDTA (ethylenediaminetetraacetic acid) tubes and stored at −20 °C until DNA isolation.

Genomic DNA was isolated from peripheral blood samples using the QIAamp DNA Blood Mini Kit (Qiagen, Hilden, Germany), according to the manufacturer’s instructions. DNA quantity and quality were assessed using a Qubit fluorometer (Invitrogen, Thermo Fisher Scientific, Waltham, MA, USA).

### 2.3. Polymerase Chain Reaction (PCR)

The coding regions of the *SLC26A9* gene (RefSeq: NM_052934.3) were PCR-amplified using specific primers (Thermo Fisher Scientific, Waltham, MA, USA) (Table 2). The primers targeted the regions in exons 3 and 17 where Luo et al. [7] identified the variants c.229G>A (p.Gly77Ser) and c.1885C>T (p.Pro629Ser), respectively.

PCR was carried out in a total volume of 17 µL, containing 2 µL of genomic DNA (10 ng/µL), 0.5 µL of each primer (10 pM), and 10 µL of NXT Taq PCR Master Mix (2x), which included NXT Taq DNA polymerase, reaction buffer, MgCl_2,_ and dNTPs. The thermocycler program consisted of an initial denaturation at 96 °C for 5 min, followed by 40 cycles of denaturation at 96 °C for 5 s, annealing at 57 °C for 5 s, and extension at 68 °C for 10 s, with a final extension at 72 °C for 1 min. PCR was performed using a TPersonal thermocycler (Biometra, Analytik-Jena, Germany).

### 2.4. Sanger Sequencing

Purified PCR products were subjected to Sanger sequencing using an automated sequencer (Applied Biosystems^®^ 3130/3130xl Genetic Analyzers, Thermo Fisher Scientific, Waltham, MA, USA) and the BigDye Terminator v3.1 Cycle Sequencing Kit (Thermo Fisher Scientific, Waltham, MA, USA). Sequence analysis was performed using FinchTV software (v1.4.0, Informer Technologies Inc., Geospiza/Digital World Biology, Seattle, WA, USA), and the resulting sequences were compared with the *SLC26A9* reference sequence (RefSeq: NM_052934.3).

## 3. Results

**Figure 1:**
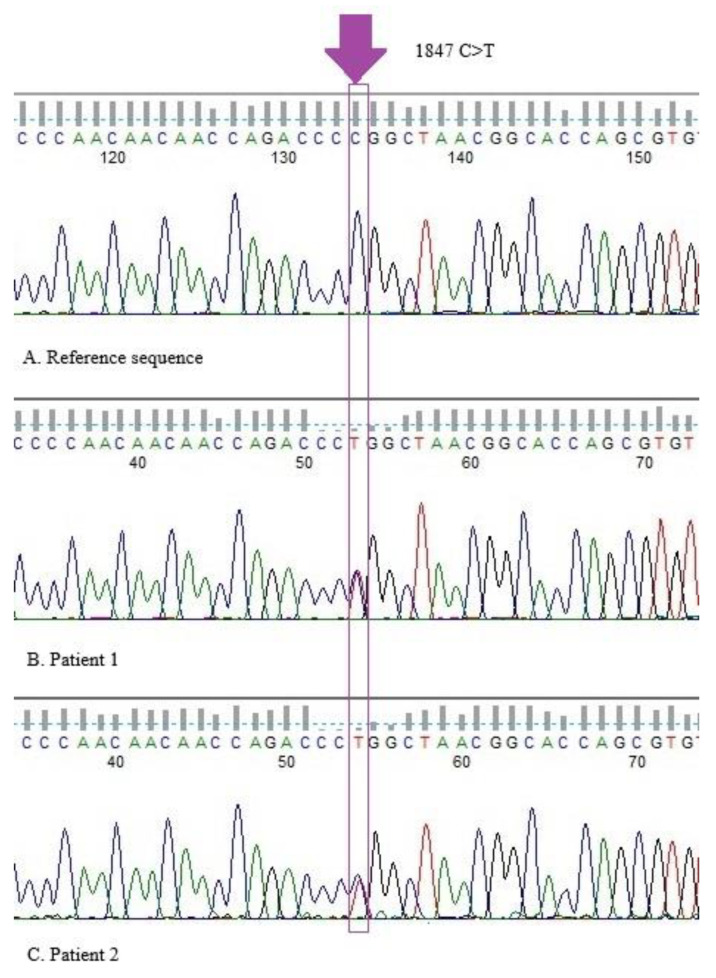
Sanger sequencing results: (**A**) reference sequence—patients without the variant; (**B,C**) sequences from two patients carrying the c.1847C>T variant.

Of the 20 patients screened, two carried a variant in the *SLC26A9* gene. The detected variant was NM_052934.4:c.1847C>T (p.Pro616Leu) in exon 17 (Figure 1), which has not been widely described in the literature and remains of uncertain clinical significance in CF. The c.229G>A (p.Gly77Ser) and c.1885C>T (p.Pro629Ser) variants reported by Luo et al. [7] were not detected in this study.

Two patients with *SLC26A9* variants were siblings carrying the p.Phe508del and c.3140-26A>G variants in the *CFTR* gene.

In the male patient, CF was diagnosed at 38 years of age. The disease manifested as mild bronchiectasis and nasal polyps, without pancreatic insufficiency. Bronchoaspirate cultures showed significant colonization with *Pseudomonas aeruginosa* and *Staphylococcus aureus*. CF diagnosis was further supported by a positive sweat test (chloride concentration 115 mmol/L; reference range < 30 mmol/L). In 2024, treatment with CFTR protein modulators (ivacaftor and tezacaftor) was initiated, resulting in partial clinical improvement without normalization of sweat chloride concentration.

The female patient was diagnosed several months after her brother at the age of 37. At the time of diagnosis, her sweat chloride concentration was 125 mmol/L. She had a history of severe asthma requiring oral corticosteroids, lower respiratory tract colonization with methicillin-sensitive *Staphylococcus aureus* (MSSA), nasal polyps, and allergic rhinitis. No bronchiectasis or pancreatic insufficiency was reported. Diagnostic evaluation confirmed the comorbidity of CF and asthma. Modification of asthma therapy and initiation of CFTR modulator treatment resulted in a reduction in CF-related symptoms, with a significant improvement in asthma control, allowing discontinuation of oral glucocorticosteroids.

Both patients received triple CFTR modulator with ivacaftor, tezacaftor, and elexacaftor in May 2025, which resulted in further clinical and spirometric improvement. However, no change was observed in the sweat chloride test, and bacterial colonization persisted, including the pathogens identified previously.

## 4. Discussion

The p.Phe508del variant in the *CFTR* gene is a class II mutation that disrupts protein trafficking to the cell membrane and impairs its function. It is the most common pathogenic variant in patients with CF and is typically associated with a severe clinical course and exocrine pancreatic insufficiency. The c.3140-26A>G variant (also known as 3272-26A>G) belongs to class V of *CFTR* variants that causes splicing abnormalities. This class is often linked to milder disease phenotypes and preserved pancreatic function [8]. Previous studies have shown that patients carrying both variants often present a milder disease course and are diagnosed later in life; however, nasal polyps are reported more frequently than in patients homozygous for p.Phe508del [6,8]. This clinical profile is consistent with the presentations of both patients in our study.

A missense variant in the *SLC26A9* gene (NM_052934.4:c.1847C>T) was identified in the present study. In silico analyses suggest a benign polymorphism; however, data on its role in diseases such as CF, where it may act as a modifier, are lacking. This variant is not reported in ClinVar. In the Franklin database, it is classified as a VUS with PM2 (pathogenic moderate) evidence based on its extremely low frequency in gnomAD population databases.

According to gnomAD v4, this variant has an allele frequency (AF) of 0.0000197 in the general population. In the GeneBe database, it is classified as “likely benign”, with PM2 (moderate) evidence due to its very low frequency in population databases with high coverage, and BP4 (strong) evidence based on computational predictions (MetaRNN = 0.041). In silico prediction tools generated the following scores for this variant: PolyPhen-2 = 0 (benign), SIFT = 0.11 (tolerated), CADD = 16 (likely benign), and MetaLR = 0.6 (damaging). The PhyloP100 conservation score for these positions is 3.59, indicating moderate conservation across mammals (Ensembl, GeneBe).

SLC26A9 (solute carrier family 26 member 9) is a multifunctional anion transporter belonging to the SLC26A family encoded by a gene comprising 21 exons. The protein functions both as an ion channel and an anion exchanger. It acts as a chloride channel mediating uncoupled chloride transport through an alternating-access mechanism, where a saturable binding site is exposed to either side of the membrane [3,9,10,11]. In addition, it facilitates chloride–bicarbonate exchange across the cell membrane [9,12]. This channel-exchanger protein is involved in physiological processes in the respiratory and gastrointestinal systems, as well as in renal function [4].

Recent identification of potentially deleterious *SLC26A9* variants in patients with rapidly progressing CF suggests that SLC26A9 may function as an alternative anion transporter in CF and a modifier associated with pulmonary phenotype severity [7].

It has been proposed that CFTR modifiers from the SLC family may participate in alternative ion transport pathways, modulate responses to therapy, and mediate protein–protein interactions and clinical presentation [13]. Co-expression analyses in lung tissue have shown that alveolar type II epithelial cells exhibit a significant co-expression pattern involving *CFTR*, *SLC6A14*, and *SLC26A9* [14].

The variant identified in our study is located in exon 17. It lies within a region that has not been structurally modeled (568–652aa) where a compositional bias is observed between residues 610 and 626. This region is part of the cytoplasmic topological domain (490–791aa). The region spanning aa 562–654 contains the STAS domain (sulfate transporter and anti-sigma factor antagonist), which includes a loop region (620–628aa) that affects SLC26A9 transporter function and contains serine- and threonine-rich regions. Amino acid substitutions in these regions may therefore influence transporter activity [3,5,15].

Bioinformatic analyses by Chen et al. (2025) [5] have demonstrated that the deletion of the entire SLC26A9-STAS domain results in loss of chloride channel function. Surprisingly, however, partial or complete deletion of the disordered STAS disordered loop significantly increases chloride transport activity [5].

The disordered loops comprise three subregions: a lysine/arginine-rich (K/R-rich) region, a central region, and an ordered S/T-rich motif. Alanine-to-serine or alanine-to-threonine substitutions in the S/T-rich motif (620–628aa) reduce SLC26A9 chloride channel activity [5]. These findings indicate that the STAS disordered loop and its subregions play a regulatory role in the function of the SLC26A9 protein. Deletion or silencing of this variable loop of human SLC26A9 may represent a novel gene therapy strategy in CF treatment [5].

It has also been suggested that SLC26A9 can interact directly with CFTR through the STAS domain of SLC26A9 and the regulatory domain of CFTR, thereby inhibiting SLC26A9 activity [16]. Variants in this region, such as p.Arg575Trp or p.Val622Met, have been described in the literature [17].

It has been hypothesized that the p.Arg575Trp variant in the *SLC26A9* gene could prevent SLC26A9-mediated functional activation of *CFTR* by altering the interaction between the two proteins. Such variants may enhance the pathogenic effect of a single *CFTR* variant and influence chloride transport across the airway epithelium through functional inhibition of CFTR [17].

Chen et al. (2012) [18] indicated that genetic variants in the *SLC26A9* gene may lead to various functional modifications of this protein, including increased or decreased activity and altered protein expression, resulting in variable clinical outcomes. The authors emphasized that assessing the genetic status of this gene is important for providing personalized care to patients with CF. In their study, the p.Val622Leu variant reduced channel transport activity by approximately 50% [18].

*SLC26A9* variants described in the literature as modifiers also include rs7512462, located in intron 5. Strug et al. (2016) [19] reported that the C allele is associated with better baseline lung function in untreated CF patients carrying “gating” *CFTR* variants (e.g., Gly551Asp). The latter authors also observed a variable response to ivacaftor in the presence of this allele, with an average improvement of approximately 25% [19]. Eastman et al. (2021) [20], however, found no association between this genotype and lung disease severity or response to ivacaftor in patients with p.Gly551Asp [20].

The rs4077468 polymorphism (intron 1, 5’UTR) is strongly associated with the risk of cystic fibrosis-related diabetes (CFRD), with the A allele linked to increased susceptibility [21]. Similarly, the A allele of rs4077469 has also been associated with a higher risk of CFRD [21].

Since the variant identified in the current study is located within a domain characterized by compositional bias, the possibilities for structural modeling of the protein and for reliable in silico prediction of the effects of amino acid substitutions are limited. Studies on the STAS domain, in which this variant was identified, have indicated its importance for proper ion transport, including CFTR-related trafficking. Alterations in this region may affect the topology of the STAS domain and, consequently, protein function. The identified variant may be benign or may modify the clinical course of CF in a protective manner. This interpretation is supported by the absence of a severe phenotype in the affected patients, whose milder clinical presentation is largely consistent with the well-documented CFTR genotype p.Phe508del + c.3140-26A>G. Further, forced expiratory volume in 1 s (FEV1) in the two patients carrying the SLC26A9 variant was 82% and 84% of the predicted value (normal range) and the mean FEV1 in the remaining patients was 54% of the predicted value.

It should also be noted that the proposed interpretation of the variant’s significance remains hypothetical, as no functional studies were performed. A study in a larger patient cohort, together with further association analyses, may enable a more detailed evaluation of the impact of *SLC26A9* variants, especially in regions with compositional bias, on disease course. Such studies may also improve understanding of the pharmacogenomic aspects of CFTR modulator therapy and its clinical efficacy, especially in the presence of additional variants in genes encoding ion channels other than CFTR.

The relatively small study cohort limits the interpretation of these findings; therefore, the results should be regarded as preliminary. Nonetheless, *SLC26A9* remains a gene of interest from both clinical and molecular perspectives. Efforts are underway to expand the study group and to broaden the scope of the analysis to the full coding sequence of the *SLC26A9* gene, which is expected to provide a more comprehensive assessment of its role in CF. Inclusion of a healthy control group would further strengthen comparative analyses. More advanced bioinformatic analyses are required to estimate the functional impact of the identified variant. This remains challenging, as the variant is located within a non-modeled region of the protein. Consequently, the proposed mechanistic interpretations remain hypothetical but provide a basis for further investigations. The present study was limited to selected regions in exons 3 and 17. Despite this restriction, an unexpected variant was identified. Future studies should include full-length sequencing of *SLC26A9* using next-generation sequencing (NGS) approaches.

## 5. Conclusions

The *SLC26A9* variant identified in the present study has not previously been reported in patients with cystic fibrosis. However, its location within the STAS domain suggests a potential role in regulating ion transport activity.

## Figures and Tables

**Table 1:** *CFTR* variants in the study population.

*CFTR* Variants		Variant Legacy Name *	Number of Patients, n (%)	Exon/Intron Number
DNA Level	Protein Level			
NM_000492.4:c.1521_1523del	NP_000483.3:p.Phe508del	F508del	12 (60)	11
NM_000492.4:c.3140-26A>G	intron	3272-26A->G	2 (10)	19
NM_000492.4:c.3909C>G	NP_000483.3:p.Asn1303Lys	N1303K	2 (10)	24
NM_000492.4:c.3454G>C	NP_000483.3:p.Asp1152His	D1152H	1 (5)	21
NM_000492.4:c.3718-2477C>T	intron	3849+10kbC->T	1 (5)	22
NM_000492.4:c.1000C>T	NP_000483.3:p.Arg334Trp	R334W	1 (5)	8
NM_000492.4:c.2012del	NP_000483.3:p.Ser670_Leu671insTer	2143delT	1 (5)	14

* According to the CFTR2 database.

**Table 2:** Primers used for Sanger sequencing of the *SLC26A9* gene.

Exons	Primer Sequence	Catalog Number
Exon 3	F: 5′ AGC AGC ATG ACT AGC TTA TGG G 3′	A15629 (Hs00729324)
	R: 5′ CTG GAA ATT TTC CCC GTT CCC T 3′	A15630 (Hs00729324)
Exon 17	F: 5′ CAT CAA GTC CAC GAA GCT GAC T 3′	A15629 (Hs00600652)
	R: 5′ CAG CAG GAC TTT GAG AAT GCG 3′	A15630 (Hs00600652)

## Data Availability

All data generated during the research work is included in the article.

## Abbreviations

CF (cystic fibrosis), CFTR (cystic fibrosis transmembrane conductance regulator), SLC26A9 (solute carrier family 26 member 9), EDTA (ethylenediaminetetraacetic acid), PCR (polymerase chain reaction), STAS (sulphate transporter and anti-sigma factor antagonist), VUS (variant of uncertain significance), NGS (next-generation sequencing), CFRD (cystic fibrosis-related diabetes), FEV1 (forced expiratory volume in 1 s).
